# Parallel profiling of antigenicity alteration and immune escape of SARS-CoV-2 Omicron and other variants

**DOI:** 10.1038/s41392-022-00910-6

**Published:** 2022-02-08

**Authors:** Cong Sun, Yin-Feng Kang, Yuan-Tao Liu, Xiang-Wei Kong, Hui-Qin Xu, Dan Xiong, Chu Xie, Yi-Hao Liu, Sui Peng, Guo-Kai Feng, Zheng Liu, Mu-Sheng Zeng

**Affiliations:** 1 State Key Laboratory of Oncology in South China, Collaborative Innovation Center for Cancer Medicine, Department of Experimental Research, Sun Yat-sen University Cancer Center, Sun Yat-sen University, Guangzhou, 510060 P. R. China; 2 Cryo-electron Microscopy Center, Southern University of Science and Technology, Shenzhen, 518000 Guangdong P. R. China; 3 Medical Laboratory of the Third affiliated Hospital of Shenzhen University, Shenzhen, 518000 Guangdong P. R. China; 4 Institute of Precision Medicine, The First Affiliated Hospital of Sun Yat-sen University, Guangzhou, 510080 Guangdong P. R. China; 5 Clinical Trials Unit, The First Affiliated Hospital of Sun Yat-sen University, Guangzhou, 510080 Guangdong P. R. China; 6 Department of Endocrinology, The First Affiliated Hospital of Sun Yat-sen University, Guangzhou, 510080 Guangdong P. R. China

**Keywords:** Infectious diseases, Infectious diseases, Structural biology

## Abstract

SARS-CoV-2 variants have evolved a variety of critical mutations, leading to antigenicity changes and immune escape. The recent emerging SARS-CoV-2 Omicron variant attracted global attention due to its significant resistance to current antibody therapies and vaccines. Here, we profiled the mutations of Omicron and other various circulating SARS-CoV-2 variants in parallel by computational interface analysis and in vitro experimental assays. We identified critical mutations that lead to antigenicity changes and diminished neutralization efficiency of a panel of 14 antibodies due to diverse molecular mechanisms influencing the antigen-antibody interaction. Our study identified that Omicron exhibited extraordinary potency in immune escape compared to the other variants of concern, and explores the application of computational interface analysis in SARS-CoV-2 mutation surveillance and demonstrates its potential for the early identification of concerning variants, providing preliminary guidance for neutralizing antibody therapy.

## Introduction

The ongoing COVID-19 pandemic threatens global public health and has already caused millions of deaths.^1–3^ The causative agent SARS-CoV-2 is a member of the beta-coronavirus family, with a single-stranded positive-strand RNA genome encoding four major structural components, the spike (S), envelope (E), nucleocapsid (N), and matrix (M) proteins.^4^ As the major glycoprotein of SARS-CoV-2, the spike protein is located on the virion membrane and is the fusion protein mediating the virus-host cell attachment, binding, and fusion process.^5^ It consists of two subunits (S1 and S2) and recognizes angiotensin-converting enzyme 2 (ACE2) on the host cell membrane as the receptor via its receptor-binding domain (RBD) within the S1 subunit, triggering a conformational change that leads to cell entry.^6–8^ Because the spike protein plays a critical role in viral infection, neutralizing antibodies against the spike protein are developed as a therapeutic approach to transiently block acute infection, and the development of a spike protein-based vaccine became the major vaccine strategy.^9–13^ Thus, any changes in the antigenicity of the spike protein of mutant variants could significantly limit the efficacy of current efforts in COVID-19 pandemic control, requiring close surveillance of concerning variants.^14^

SARS-CoV-2 has a high intrinsic rate of mutation due to its single-stranded RNA genome and beneficial mutations that alter critical amino acid residues at specific sites and further change the molecular dynamics of protein–protein interactions to allow immune escape can quickly spread in the vaccinated population.^15–18^ The spike protein plays a unique role in viral infection, and thus its mutations have become a major research focus.^19^ The first broadly-noticed spike mutation D614G was reported to result in a moderate increase of transmissibility, which was further elucidated by a recent structural analysis of the impact of spike mutations, highlighting the crucial role of detailed structural profiling of mutation-driven antigenic drift.^20–24^

As the pandemic persisted and increasing numbers of SARS-CoV-2 variants have been identified, the greater concern is given to multiple widely-spread emerging variants bearing critical mutations and displaying heterogeneous resistance to vaccine-elicited sera.^25,26^ Variants of concern (VOC) classified by the World Health Organization (WHO) include the alpha, beta, gamma, and delta strains, which exhibit increased transmissibility, can lead to severe disease, and reduce the neutralizing ability of antibodies, especially vaccine-elicited sera, bringing great challenges for achieving population-level immunity and clinical management of infected cases.^27–29^ The alpha variant, also termed B.1.1.7, first emerged in the United Kingdom and spread worldwide. Its spike protein contains several mutations and is distinguished by the N501Y substitution located in the RBD, which affects spike interactions with ACE2 and neutralizing antibodies, leading to decreased neutralization capability and vaccine resistance.^30,31^ Then subsequently discovered beta (B.1.351) and gamma (P.1) variants also contain the N501Y substitution, respectively harbor additional mutations at K417 and E484, which further increased their resistance to antibodies vaccine-elicited sera.^32–37^ After May 2021, the delta variant (B.1.617.2) became the major VOC, displaying a rapid worldwide spread. It is distinguished from the other three variants by the two unique mutations L452R and T478K located in the RBD region, which also impacted the variant’s sensitivity to antibodies or vaccine-elicited sera.^38–40^ In the meantime, the Kappa variant (B.1.617.1)^41^ and the lambda variant (C.37)^42^ were found with a mutation at L452, E484, and F490 and were listed as a variant of interest (VOI) by WHO. Recently, Omicron variant (B.1.1.529),^43,44^ as a newly emergent strain of SARS-CoV-2, drew global attention due to its rapid spread and unprecedented complexity in mutation patterns. It harbored 30 different mutations at spike protein, with 12 located in the RBD region, making it more unpredictable in antigenicity alteration and immune escape.

Recent progress in computational prediction of protein structures such as AlphaFold2 brought unprecedented insights into previously unrevealed protein structures.^45^ Computational interface analysis or evaluation also brought unique advantages in drug screening, antibody affinity maturation, and protein optimization and has shown great potential for explaining the impact of mutations on the binding of spike protein to ACE2.^46–49^ The application of such methods for early surveillance of SARS-CoV-2 mutations may help estimate the effect of mutation-driven immune escape and adaptation during the clinical application of antibody-based antiviral therapies.

Our study performed both computational and experimental profiling of mutations in various SARS-CoV-2 variants in parallel. The computational interface analysis provided preliminary evidence of affinity changes induced by critical mutations further confirmed through analysis of in vitro binding kinetics and pseudovirus assay. Computational analysis of mutations in combination with in vitro study could be valuable for the surveillance of SARS-CoV-2 variants, and that mutations found in each variant might play heterogeneous roles during the molecular interaction with neutralizing antibodies or the cellular receptor, leading to diverse immune evasion mechanisms.

## Results

### Interface analysis of SARS-CoV-2 RBD mutations

Various computational modeling algorithms for protein–protein interface analysis have been developed, providing alternative approaches for large-scale screening of potential targets and high-throughput evaluation of mutation candidates, enabling accurate prediction of the structural effects of specific mutations. Therefore, we selected a panel of neutralizing antibodies with available RBD-bound crystal structures, including S2-E12, Regdanvimab (or CT-P59), Regn10987, Regn10933, P2B-2F6, Fab2-15, S2-M11, S2-H14, COVA1-16, CB6, and CR3022,^9,50–57^ and selected a high-resolution ACE2-RBD complex structure for interface analysis.^8^ To achieve a rapid and accurate assessment of the interface after mutation, we used FlexddG,^58^ developed based on the Rosetta macromolecule modeling suite, for interface evaluation of mutations from SARS-CoV-2 variants located in the RBD, as this region is the key target for virus neutralization due to the underlying ACE2-binding interface (Supplementary Fig. 1).

**Fig. 1:**
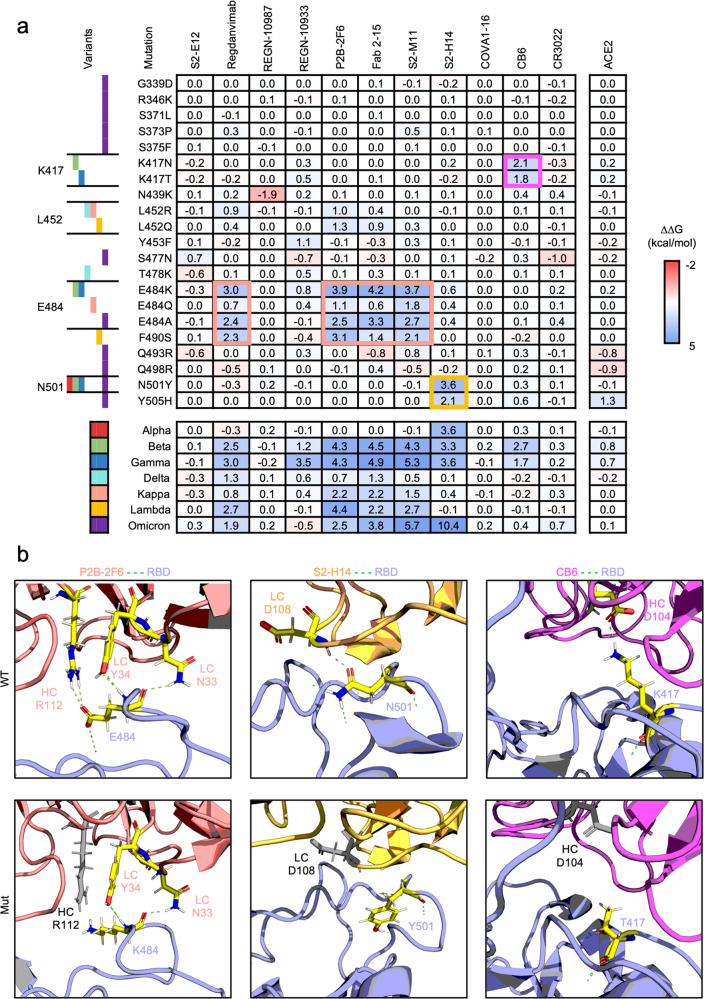
Computational interface analysis of SARS-CoV-2 spike protein variants by FlexddG. **a** Interfacial free energy (∆∆G) heatmap for complexes of neutralizing antibodies or ACE2 with RBD variants containing the indicated mutations. The ∆∆G values are shown in the table. **b** Representation of the structural interface of the antibodies P2B-2F6, S2-H14, and CB6 with wild-type or mutated RBD. The interacting residues are shown as sticks and colored by atom (carbon: yellow, hydrogen: white, nitrogen: blue, oxygen: red). The hydrogen bonds are displayed as green dashed lines. Residues in antibodies that lost hydrogen bonds to the RBD due to the indicated mutations are gray. The shown ΔΔG of each antibody or receptor in contact with the indicated mutation of the RBD is the average value from 35 output structures generated by flexddG

We first collected and preprocessed the co-crystal structures of the neutralizing antibody or receptor with wild-type SARS-CoV-2 RBD as the input structure for interface analysis. By specifying mutations from different SARS-CoV-2 variants, the ∆∆G value reflecting the interfacial free energy state was calculated compared to the unmodified status and could offer a reference for evaluating the change of binding affinity caused by each mutation (Fig.  1a). An absolute value of ∆∆G over 1 (|∆∆G | ≥ 1 kcal/mol) was regarded as a significant change in interfacial free energy.

Among the variants, K417, L452, E484, and N501 located at the RBD region were most affected, and mutation at these critical residues exerted heterogenous influence on the neutralizing antibody binding capability. It was shown that mutation at E484 in the Beta, Gamma, Kappa, and Omicron variant was identified as the critical mutation undermining the binding affinity because most neutralizing antibodies targeting RBD displayed a significant increase in ∆∆G, and it seemed that F490S from the Lambda variant showed similar effect as mutation at E484. Furthermore, mutation at K417 and N501 also displayed potentially diminished binding affinity to specific antibodies (CB6 and S2-H14). The mutation at L452 did not significantly change ∆∆G like the single point mutations from other VOC strains. Moreover, it was found that all mutations barely affected the affinity with ACE2.

Then, we performed a further combinatorial investigation in full mutation sets from SARS-CoV-2 variants. The Omicron variant combined the characteristics brought by mutation at E484 and N501, resulting in a decrease in affinity with Regdanvimab, P2B-2F6, Fab 2-15, and S2-M11 sensitive to E484 mutation, and S2-H14 sensitive to N501 and Y505 mutation. For Beta and Gamma variants even harboring the mutation at K417, the affinity with CB6 was also decreased due to the sensitivity to K417 mutation, possibly suggesting that Beta and Gamma variants displayed an even broader resistance to current antibodies. Nevertheless, it deserves to point out that most Omicron variant mutations did not influence antibody interaction in our antibody panel.

Unlike the antibodies, the ACE2 interface ∆∆G was almost unaffected by both single mutation and mutation set from variants located on RBD, indicating that the mutations found in SARS-CoV-2 VOC possibly underwent delicate selection to avoid reduction infectivity.

### Structural exploration of the mutational impact on RBD-antibody interface

To further explore the detailed impact of mutations on the antibody-RBD interface, we compared the input wild-type structure after relaxation and the output mutant structure generated by flexddG with the lowest structural energy state. Although different neutralizing antibodies (nAb) displayed heterogenous vulnerability to mutations, a significant ∆∆G increase indicated a similar molecular mechanism, discovered by carefully examining the co- structures of P2B-2F6, S2-H14, and CB6 with RBD (Fig. 1b). Specifically, residue E484 of the RBD interacts with the N33 and Y34 of the light chain and R112 of the heavy chain of the P2B-2F6 antibody, and when the residue was mutated to K484, it excluded R112 from the polar interaction network and diminished the H-bond interaction with Y34. Similar to P2B-2F6, other antibodies, including Regdanvimab, Fab 2-15, and S2-M11, also interacted with E484 in the RBD, and the interaction was impaired after mutation K484 (Supplementary Fig. 2). Likewise, the interaction between N501 of the RBD and D108 of the light chain of S2-H14 and the interaction between K417 of the RBD and D104 of the heavy chain of the CB6 antibody was absent after certain mutations. These results not only suggested that flexddG was sensitive enough for the evaluation of mutations undermining polar interactions but gave structurally-rational support for the interfacial ∆∆G estimation after specifying the mutation.

**Fig. 2:**
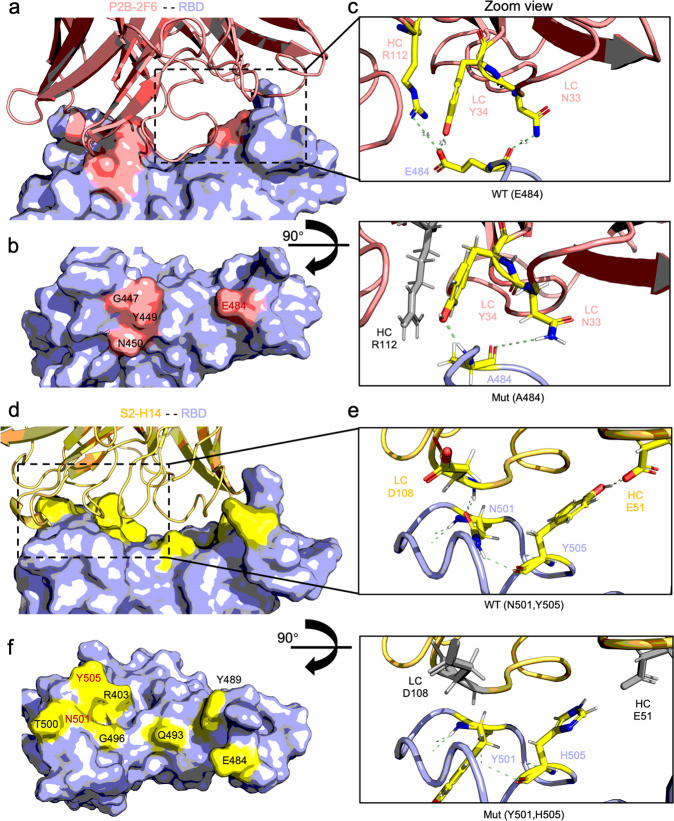
Structural overview of E484A and N501Y/Y505H mutational impact on P2B-2F6 and S2-H14 antibody interaction of Omicron variant. **a** Overview of P2B-2F6 complexed with RBD (PDB: 7BWJ). The P2B-2F6 is presented in a red cartoon, and the RBD is presented on a light blue surface with a red footprint. **b** The footprint of key residues on RBD interacting with P2B-2F6. The key residue names are marked beside, and the mutated residue E484 is colored in red. **c** Zoom view of wild type (E484) and mutant (A484) structure of RBD with P2B-2F6. Critical residues participating in the interaction between RBD and P2B-2F6 are presented in the sticks, and the polar bond is presented as dashed green lines. For mutant structure, the residue from antibody excluded from the polar interaction due to A484 mutation is colored in gray. **d** Overview of S2-H14 in complex with RBD (PDB:7JX3). The S2-H14 is presented in a yellow cartoon, and the RBD is presented on a light blue surface with a yellow footprint. **e** The footprint of key residues on RBD interacting with S2-H14. The key residue names are marked beside, and the mutated residues N501/Y505 are colored in red. **f** Zoom view of wild type (N501/Y505) and mutant (Y501/H505) structure of RBD with S2-H14. Critical residues participating in the interaction between RBD and S2-H14 are presented in the sticks, and the polar bond is presented as dashed green lines. For mutant structure, the residues from antibody excluded from the polar interaction due to Y501/H505 mutation are colored in gray

Further structural investigation on the Omicron revealed more detailed information on how E484A and N501Y/Y505H drove the antigenicity alteration (Fig. 2). Similar to E484K, E484A would exclude R112 of the heavy chain of P2B-2F6 from the polar interaction network, destabilizing the interface between RBD and antibody. However, for S2-H14, the N501Y/Y505H synergized the diminished polar interaction. The simultaneous mutation in the two sites completely invalidated the interaction between RBD N501 with D108 of the light chain and RBD Y505 with E51 of the heavy chain of S2-H14, which may explain the increase in ∆∆G estimation in comparison with N501Y solely.

As L452R and T478K did not significantly impact the antibody interface ∆∆G like other mutations, we further inspected the mutant structure rendered by flexddG. Indeed, we found that Regdanvimab and P2B-2F6 did not establish polar contacts with the RBD at L452. However, the mutation L452R still negatively influenced the interfacial stability of the two antibodies (Supplementary Fig. 3). As a non-polar amino acid (AA), L452 could maintain a loose but relatively close distance to I103 and V105 from the heavy chain of P2B-2F6 in a non-polar cluster. The conversion to the positively charged R452 could lead to a transition to a polar residue surface on the side of RBD, repelling the non-polar AA from the antibody side, potentially causing a decrease in interfacial complementarity and stability. In comparison, Y106 and R107 from the heavy chain of Regdanvimab close to the L452 from the RBD were polar AA, causing a similar local polar/non-polar interface as R452 with P2B-2F6. However, the mutant R452 introduced a new positively charged residue in the RBD, resulting in emergent polar exclusion between R452 on the RBD and R107 on the antibody, leading to destabilization.

The flexddG algorithm displayed high performance in estimating the impact of mutations of SARS-CoV-2 variants on the antibody binding capacity. The ∆∆G results precisely reflected the changes of interfacial interaction after mutation at a certain site, and different mutations resulted in heterogeneous interactions between RBD and the antibody, leading to different extents of interfacial free energy ∆∆G change and binding affinity decrease. The high sensitivity of the interface analysis could be helpful for early assessment of the effects of SARS-CoV-2 mutations on RBD. Additionally, the flexddG results revealed that mutations from SARS-CoV-2 variants diminished neutralizing antibody binding to varying degrees, which may be strongly correlated to their behavior in immune escape.

### In vitro binding profiles of SARS-CoV-2 variants with neutralizing antibodies

To determine the binding capability of SARS-CoV-2 spike variants with antibodies or receptor ACE2, we purified different spike protein variants and neutralized antibodies and performed a complete kinetic analysis to investigate the mutational impact on the binding ability. To provide an experimental reference for in vitro antibody studies, we included two additional RBD-specific antibodies without available co-crystal structures with RBD, called COV2-2196^59^ and IgG1-ab1,^60^ as well as the NTD-specific antibody 4A8^61^ to avoid over-interpretation.

**Fig. 3:**
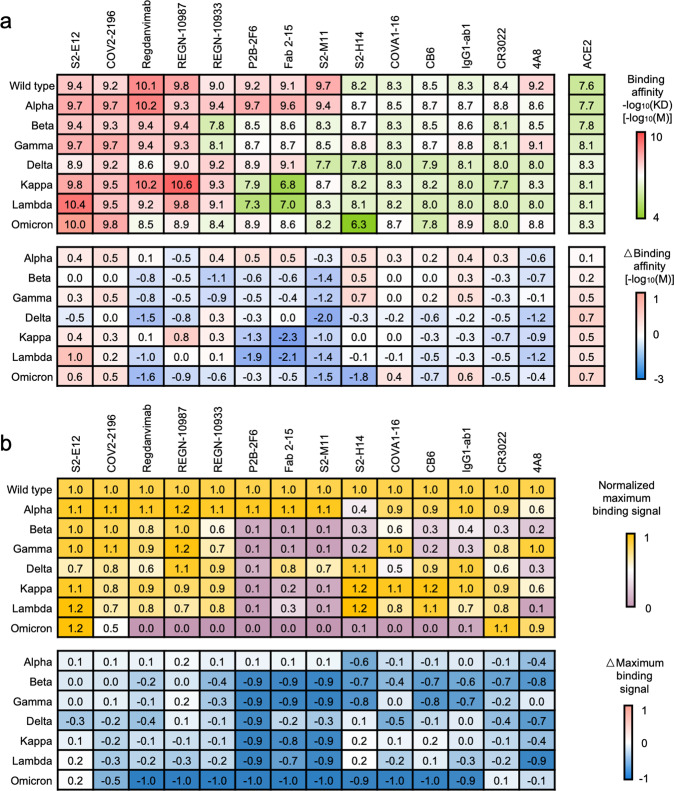
Kinetic profile of the SARS-CoV-2 variants determined by biolayer interferometry. **a** Binding affinity heatmap of SARS-CoV-2 spike variants with neutralizing antibodies and ACE2. The binding affinity is presented as a heatmap colored in a red-to-green gradient. The binding affinity −log_10_(*K*_D_) [−log10(M)] value is listed in the table. A deeper red represents a higher affinity of the indicated antibody to the indicated spike protein or RBD variant, and a deeper green represents a lower affinity. The change of binding affinity (ΔBinding affinity) [−log10(M)] due to mutations in the spike protein or RBD is presented as a heatmap colored in a pink-to-blue gradient. A deeper pink represents a larger increase in affinity than the wild type, and a deeper blue represents a larger decrease in affinity. The binding affinity of each antibody for the indicated spike protein or RBD variants was assessed based on the calculated equilibrium dissociation constant (*K*_D_) in the kinetic assay, based on a global fitting model of six different concentration curves. **b** Normalized maximum binding signal heatmap of SARS-CoV-2 spike variants with neutralizing antibodies. The maximum binding signal affinity is presented as a heatmap colored in a yellow-to-purple gradient. The maximal binding of 200 nM wild-type spike protein with each antibody is used as the control signal. The ratio of the maximal binding signal of each spike variant to the control signal is calculated as the normalized maximal binding signal. A deeper yellow represents the indicated antibody’s stronger maximal binding ability to the indicated spike protein, and a deeper purple represents a lower ability. The change of binding signal (ΔBinding signal) due to mutations in spike protein is presented as a heatmap colored in pink-to-blue gradient. A deeper pink represents a larger increase of maximal binding ability than the wild type, and a deeper blue represents a larger decrease in binding capability

An overview of the kinetic profiling of SARS-CoV-2 variants revealed similar trends in computational interface analysis (Fig. 3a). It was shown that all SARS-CoV-2 variant spike proteins manifested diminished constant binding affinity to various antibodies. Inconsistency to flexddG result, Omicron, Beta, Gamma, Kappa, and Lambda variants with a mutation at E484 displayed the most potency in resistance to binding of nAbs such as REGN-10933, P2B-2F6, Fab 2-15, and S2-M11. Although Regdanvimab maintained a relatively high binding affinity with mutant spike proteins, the variants with mutations at E484 still reduced its binding affinity. As hypothesized, S2-E12 were not affected by the mutations and sustained a comparable affinity for wild-type spike protein and different variants. For the delta variant, although Regdanvimab and P2B-2F6 were less affected by L452R than by E484K/A/Q according to the flexddG results, we found that their affinity for the delta spike protein variants was significantly decreased, indicating that other mutations beyond the RBD may influence antibody binding or that flexddG may underestimate its impact on antibody binding. Despite extensive impacts on the binding of neutralizing antibodies by mutations, the receptor binding capability of all tested spike variants was barely affected, suggesting a delicate positive selection during the SARS-CoV-2 mutation promoting immune escape without sacrificing infectivity and this phenomenon was also observed in the computational interface analysis.

In addition to *K*_D_ reflecting the balanced binding status, the maximum binding signal can help understand the maximal binding capability of certain interacting pairs and correct the overfitting result of *K*_D_ under extremely low binding signal. In this study, the maximal antibody saturation of SARS-CoV-2 spike variants was analyzed. By extracting the maximal binding signal of each antibody with spike variants at 200 nM, we calculated the normalized maximal binding signal of each nAbs with different spike protein variants (Fig. 3b and supplementary Fig. 4). The Omicron variant spike was barely bound to almost all antibodies. Beta, Gamma, Kappa, Lambda with a mutation at E484 or F490 maintained the diminished binding capability P2B-2F6, Fab 2-15, and S2-M11. Except for the E484, Beta and Gamma variant spike with a mutation at K417 and N501Y exhibited low binding capability to S2-H14 and CB6 as estimated by flexddG.

To understand the impact of mutations on antibody binding, we directly incubated the alpha and gamma variant spike proteins with antibodies or ACE2 to investigate efficiency changes in antigen-antibody or antigen-receptor complex assembly (supplementary Fig. 5). According to kinetic assays, both alpha and gamma variant spike proteins could form stable complexes with ACE2, S2-E12, and COV2-2196, as their binding was little affected by mutations. However, the gamma variant spike bearing the E484K mutation could not form a stable complex with P2B-2F6, as shown by SDS-PAGE analysis and size-exclusion chromatography.

The kinetic profiles of the SARS-CoV-2 variants were highly consistent with the interface analysis result. Spike protein of variants with shared mutation displayed a similar response to antibody binding, which the flexddG could accurately predict at certain mutation sites. However, the unexpected low binding of Omicron variant spike to all the antibodies may indicate that other mutations without clear signs on flexddG may participate in the disruption of RBD-antibody interaction. The low binding to antibodies would determine its resistance to neutralization. Besides, we could also observe that the constant binding affinity may not be an ideal indicator for binding status determination in a parallel study on SARS-CoV-2 spike protein variants. The normalized maximum binding capability may be better for surveillance of antigenicity alteration of the SARS-CoV-2 spike proteins. All in all, the high consistency between computational interface analysis, structural inspection, and kinetic analysis revealed that mutational impact on RBD-antibody interface undermines the interaction in between at atomic-scale, resulting in decreased binding capability.

### Immune escape profiling of SARS-CoV-2 variant pseudoviruses from neutralizing antibodies

**Fig. 4:**
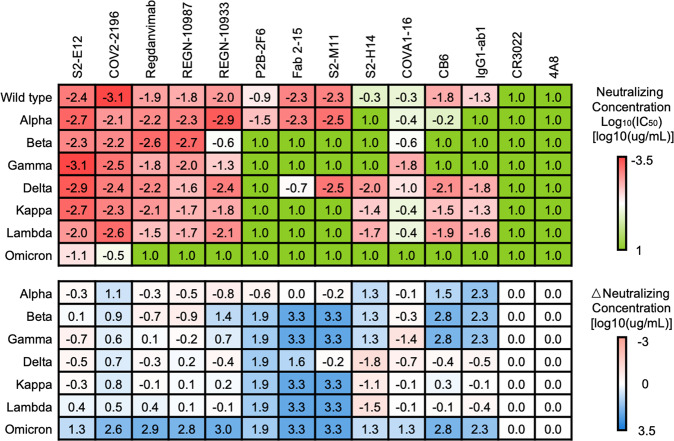
Immune escape profile of SARS-CoV-2 variants determined by pseudovirus neutralization assay with the indicated antibodies. The antibody neutralization efficacy is presented as a heatmap colored in a red-to-green gradient. The neutralizing concentration log_10_(IC_50_) [log_10_(µg/mL)] value is listed in the table. A deeper red represents a stronger neutralization ability of the indicated antibody for the pseudovirus expressing the indicated spike protein, and a deeper green represents a lower neutralization ability. The change of neutralization efficacy (∆neutralizing concentration) [log_10_(µg/mL)] due to mutations in the spike protein is presented as a heatmap colored in a pink-to-blue gradient. A deeper pink color represents a larger increase in neutralization ability than the wild type, and a deeper blue represents a larger decrease in neutralization ability. The IC_50_ values of each antibody toward the indicated pseudoviruses shown in the heatmap are the average results from duplicate experiments

After confirming the changes of antigenicity of SARS-CoV-2 variants by in vitro binding assays, we further evaluated the resulting immune escape effect in a pseudovirus neutralization assay (Fig. 4 and supplementary Fig. 6). We produced pseudoviruses bearing wild-type or mutant spike proteins harboring all mutations from the SARS-CoV-2 variants and determined the neutralizing IC_50_ of the antibodies by measuring the intracellular luciferase reporter signal after using different concentrations of neutralizing nAbs to block pseudovirus infection.

The neutralization efficacy was highly correlated with the binding kinetics, as the antigen-binding capability is one of the fundamental factors for SARS-CoV-2 antibody neutralization. Most antibodies maintained their efficacy with the Alpha and Delta variants, while the majority could not block the Omicron, Beta, Gamma, Kappa, and Lambda variants, keeping consistency with the kinetic assay. It was still observed that the Omicron variant displayed the strongest resistance to almost all antibodies, as reported by the kinetic assay. Only S2-E12 and COV2-2196 could neutralize the infection of the Omicron variant in a somehow weakened manner. However, for other variants, computational interface analysis showed remarkable performance in predicting the potential impact on antibody efficacy due to that mutation at E484 affecting Regdanvimab, P2B-2F6, Fab 2-15 and S2-M11, at K417 affecting CB6 and at N501 or Y505 affecting S2-H14 all simultaneously largely increase the interfacial ∆∆G and significantly reduce the neutralizing activity.

The pseudovirus neutralization assay provided functional evidence for mutations driving immune escape of the SARS-CoV-2 variants. The consistency of kinetic and immune escape characteristics of both the complete mutation set derived from the variants highlighted that interruption of the antibody-antigen interaction at the atomic scale, ascribed to mutations at single residues, determined the fate of antibody neutralization efficiency. Although the general neutralization efficiency of each antibody for SARS-CoV-2 variants could be similar, the molecular mechanism for antibody resistance showed great diversity among the different variants, indicating that accurate profiling of the SARS-CoV-2 variants at single-residue resolution by both computational and experimental methods will be critical for a detailed investigation of the mechanisms of antibody resistance. Finally, these approaches will be integral to further surveillance of the impact of emerging mutations on the currently available antibody-based therapies.

## Discussion

The SARS-CoV-2 variants have become a major obstacle in controlling the COVID-19 pandemic. As the spike protein plays an essential role in receptor recognition and cell entry, a mutation on the spike protein can lead to critical alterations in antigenicity and subsequent immune escape. The RBD is the immune-focused region of the spike protein, which directly interacts with the receptor ACE2 and targets most neutralizing antibodies. Therefore, close surveillance of spike protein mutations on the RBD is critical for evaluating the mutation-induced antigenicity drift and immune escape of emergent SARS-CoV-2 variants. Currently, the five SARS-CoV-2 variants Alpha (B.1.1.7), Beta (B.1.351), Gamma (P.1), Delta (B.1.617.2), and the newest Omicron (B.1.1.529) have been identified as variants of concern (VOC). Although not listed as VOC, some circulating variants, such as Kappa (B.1.617.1) and Lambda (C.37), displayed a high spread rate. There is increasing evidence that these variants have higher transmissibility, cause more severe disease, and, more importantly, reduce the neutralization efficacy of antibodies isolated from the convalescent population or generated after vaccination. Therefore, a detailed investigation of mutations harbored by these variants was required to reveal the molecular mechanism of their changed antigenicity and immune escape.

Recent advances in computational macromolecule modeling provided a new approach for quantitative evaluation of protein–protein interactions, facilitating drug development and antibody affinity maturation. By virtual screening of specific parameters based on available structure information, rapid and accurate in silico assessment of large-scale mutation libraries or protein modifications can be achieved, which was ideal for an initial exploration of the effects of the emergent SARS-CoV-2 mutations. In this study, flexddG was used to evaluate the mutations of variants at the single-residue resolution, and it exhibited high sensitivity in estimating the mutation-induced changes of protein–protein interactions. Disruption of polar contacts would lead to more significant changes in interfacial ∆∆G than the disruption of non-polar interactions, but flexddG could still detect small increases of instability due to the introduction of polar exclusion to existing polar residues or transition of the polar interface to a non-polar residue, which could also lead to dramatic alterations of the antibody binding capability.

The computational analysis observed that mutations from different variants had diverse effects on antibody binding, and mutation E484 seemed to be the most critical mutation, largely diminishing antibody affinity to spike protein, consistent with the results of kinetic and pseudovirus neutralization assays, which may explain the reason for the significant immune escape exhibited by variants that carry this mutation. However, it should be noted that the resolution of input structure can affect the reliability of flexddG results, which deserves further investigation in the future and could not be fully considered within the scope of this study. It should be pointed out that the vast majority of mutations from the Omicron variant displayed little influence on antibody binding as detected by flexddG, unlike other variants. The mutations at G339, R346, S371, S373, and S375 located on the side of RBD may not directly participate in interaction with antibody but could alter the conformation of RBD through overall structural impact, which cannot be revealed by flexddG and but could be further explored by kinetic assay and neutralizing assay.

Using kinetic assays and an in vitro assembly trial, we acquired a more detailed binding profile for the SARS-CoV-2 variants with a panel of neutralizing antibodies and ACE2. The results of the kinetic assays were generally in agreement with the computational interface analysis, suggesting that the molecular interactions at the atomic level could determine protein–protein interactions at large. Almost all increases of calculated ∆∆G were accompanied by lower *K*_D_ in antibody binding. Unexpectedly, the maximal binding capability was identified as the critical factor for evaluating alterations of antibody binding. Although S2-H14 and CB6 displayed little change in *K*_*D*_ value when confronted with the mutations at N501 and K417 during binding to the spike protein, their maximal binding capability was significantly reduced.

Furthermore, Regdanvimab partially preserved its maximal binding to the spike protein with the L452R mutation but displayed a dramatic decrease of *K*_D_ in the kinetic assay. These findings indicate that different mutations have a distinct influence on the binding characteristics of different antibodies, with diverse impacts on neutralization efficacy. Further pseudovirus neutralization assays proved this result. One mutation, in particular, was able to diminish antibody binding broadly, and E484 mutation-driven antibody resistance was observed in various variants. However, the preservation of *K*_D_ values during the binding to spike protein variants with N501Y or K417T/N mutations did not ensure the effectiveness of S2-H14 or CB6 in the neutralization assay.

In contrast, Regdanvimab was still effective in neutralizing pseudovirus with the L452R mutation despite its low *K*_D_ value in the kinetic assay. Maximal binding was the determinant of effectiveness for these antibodies, and those with a higher maximal binding capability appear to be less sensitive to certain mutations causing a drastic change in the *K*_D_ value. Therefore, a more cautious conclusion for antibody effectiveness should be made based on the kinetic analysis, and a detailed assessment of maximal binding should be included in this consideration.

A recent study revealed that applying a cocktail of available neutralizing antibodies could significantly improve the neutralization efficacy by targeting diverse binding epitopes.^62^ Since in our study, we noticed that the 14 extensively studied antibodies displayed heterogeneous sensitivity to different mutations. We believe that an appropriate combination of antibodies with different vulnerabilities to specific mutations could still result in effective inhibition of SARS-CoV-2 variant infection synergistic activity. This computational analysis offers a valuable reference for matching primary antibodies for such application scenarios, avoiding the need for slow and expensive large-scale random in vitro screening.

Our study systematically explored the possibility of performing parallel profiling of the antigenicity changes and immune escape of Omicron and other SARS-CoV-2 variants by computational analysis and in vitro binding assays with a panel of neutralizing antibodies. It was shown that Omicron exhibited significant neutralizing escape from most available antibodies. Besides, we found that interface analysis was effective in the preliminary estimation of mutational impact on antibody binding. The in-silico methods could identify critical mutations with a high possibility of causing diminished in vitro binding capability, and impaired neutralizing activity was confirmed through in vitro kinetic assays.

## Materials and methods

### Computational interface analysis

We used flexddG to detect the impact of mutations on the affinity of the interface between the RBD and receptor ACE2 or neutralizing antibodies. FlexddG is a program developed to evaluate protein–protein interfaces based on the Rosetta modeling suite.^58^ It uses “backrub” to generate a collection of wild-type or mutant models. By torsion minimization, side-chain repacking and averaging of model energy, the interface ΔΔG was calculated to determine the alteration of interfacial free energy and further assist in evaluating the affinity of the designated protein complex after the specified mutation. Firstly, we used Rosetta FastRelax to preprocess the input structures, and for each protein complex, the structure with the lowest energy out of the 15 relaxed models was selected for the next step. Then, FlexddG was used to calculate the ΔΔG of the relaxed structure with the specified mutation. The average ΔΔG value of 35 models generated by flexddG was used as the final result for each structure with a given mutation. To display the protein complex interface, the relaxed structure with the lowest energy was selected as the WT structure, and the mutated structure with the lowest energy generated by flexddG was selected as the Mut structure.

### Plasmid construction

The sequences encoding spike (S) protein with a 19 amino acid deletion at the C-terminus from SARS-CoV-2 wild type (GenBank: MN985325.1) Alpha variant/MILK-9E05B3/2020 (Lineage: B.1.1.7; GISAID accession ID: EPI_ISL_601443), Beta variant/NHLS-UCT-GS-1067/2020 (Lineage: B.1.351, GISAID accession ID: EPI_ISL_700428), Gamma variant/IC-0561/2021 (Lineage: P.1; GISAID accession ID: EPI_ISL_792680), Delta variant/MP-NCDL-2509230/2020 (Lineage: B.1.617.2; GISAID accession ID: EPI_ISL_2461258), Kappa variant/ CNRST-IND2-2021/2021 (Lineage: B.1.617.1, GISAID accession ID: EPI_ISL_1719097), Lambda variant/ UPCH_cov0463/2021 (Lineage: C.37, GISAID accession ID: EPI_ISL_2158693) and Omicron variant/Rega-20174/2021 (Lineage: B.1.1.529, GISAID accession ID: EPI_ISL_6640916) were codon-optimized and synthesized (GenScript). The single-mutation variants of the spike protein based on the wild type were generated by PCR using the QuickChange site-directed mutagenesis kit (Vazyme, C113-01) following the manufacturer’s instructions. The plasmids encoding wild-type or variant spike protein fused with an N-terminal Kozak sequence and C-terminal 3X flag tag were cloned into the mammalian expression vector pCMV14 using the CE-II cloning system. The wild type and variants of SARS-CoV-2 HexaPro constructs (residue 16-1138) were produced as previously reported.^63^ Briefly, the four synthetic spike genes were used for pseudovirus production as a PCR template to generate the HexaPro constructs with proline residues substituting F817, A892, A899, A942, K986, and V987, the GSAS amino acid sequence replacing the furin cleavage site (residues 682-685), the addition of a flexible linker (GSAS), and a T4 foldon trimerization motif at the C-terminus. The sequence encoding the RBD of the SARS-CoV-2 prototype (wild type spike residues 319-541) was also synthesized (GenScript). The monomeric hACE2 (residue 19–615) was produced as previously described.^64^ All SARS-CoV-2 HexaPro spike protein(*59*) used for protein production were fused with a tissue plasminogen activator (TPA) signal at the N-terminus, HRV3C protease recognition site, octa-histidine tag, and Twin-Strep-tag at the C-terminus, and then cloned into the mammalian expression vector VRC8405 (gifted by Dr. Gary J. Nabel).

The heavy and light chain sequences of 14 potent neutralizing monoclonal antibodies (mAbs), including REGN-10933, Regdanvimab, S2-E12, COVA1-16, S2-H14, S2-M11, CB6, IgG1-ab1, P2B-2F6, CR3022, COV2-2196, 4A8, Fab 2-15, and REGN-10987 tested in this study were retrieved from the National Center for Biotechnology Information (NCBI) and Protein Data Bank (PDB, codon-optimized, synthesized (GenScript), and cloned into the antibody expression vector.

### Protein expression and purification

Plasmids encoding SARS-CoV-2 HexaPro and hACE2 were mixed with polyethylenimine (Polysciences, Cat# 24765) at a weight ratio (w:w) of 1:3 in serum-free Union 293 medium and used to transiently co-transfect suspension Expi293F cells. After six days, the cell culture supernatant was harvested by centrifugation, filtered through a 0.22 µm pore-size vacuum membrane, and applied to Ni Sepharose excel resin (Cytiva, Cat# 17371201). For the purification of SARS-CoV-2 HexaPro wild type and variants, the resin was washed with a buffer composed of 50 mM HEPES pH8.0, 300 mM NaCI, 30 mM imidazole, 5% glycerol, and 0.02% NaN_3_, and eluted with a buffer composed of 50 mM HEPES pH8.0, 300 mM NaCI, 500 mM imidazole, 5% glycerol, and 0.02% NaN_3_. For the purification of SARS-CoV-2 RBD wild type, its variants, and hACE2, the resin was washed with a buffer composed of 50 mM HEPES pH 7.4, 300 mM NaCI, 30 mM imidazole, 0.02% NaN3, and eluted with a buffer composed of 50 mM HEPES pH 7.4, 300 mM NaCI, 500 mM imidazole, and 0.02% NaN_3_. The eluted proteins were concentrated using 10 kDa MWCO Amicon Ultra centrifugal filters (Merck Millipore, Cat# UFC901096). The protein of interest was further purified by size-exclusion chromatography using a Superose 6 Increase 10/300 GL column (Cytica, Cat# 17517201) in phosphate-buffered saline (PBS, pH 7.4), aliquoted, and stored at −80 °C until further use.

To produce the nAbs as described above, plasmids encoding the heavy and light chain were used at a weight ratio (w:w) of 5:6 to co-transfect suspension Expi293F cells using polyethylenimine as above. After 5 days, the cell supernatant containing mAbs was harvested, loaded onto protein A resin (GenScript), and eluted with glycine buffer at pH 3.0. The antibodies were further purified by size-exclusion chromatography using a Superose 6 Increase 10/300 GL column in PBS, pH 7.4. Finally, the antibodies were aliquoted and stored at −80 °C. Protein concentrations were measured using the BCA method.

### Cell lines

HEK293T cells were obtained from ATCC (CRL-3216) and cultured in Dulbecco’s minimal essential medium (DMEM) supplemented with 10% heat-inactivated fetal bovine serum (FBS) and 1%(v/v) penicillin-streptomycin. Human angiotensin-converting enzyme 2 (ACE2) stable-expressing HEK293T cells (hACE2-HEK293T) were derived from HEK293T cells by transduction with a lentiviral vector encoding the human ACE2 gene. Suspension Expi293F cells were obtained (ThermoFisher, Cat# A14527) and grown in serum-free Union 293 medium (Union, Cat# UP1000) with shaking at 120 rpm and 37 °C in a humidified atmosphere comprising 5% CO_2_. All cell lines in this study were confirmed to be free of mycoplasma contamination using MycAway™ Treatment (1000×) Mycoplasma Elimination Reagent (Yeasen, Cat# 40607ES03).

### Protein quantification and storage

The protein concentrations were determined using a NanoDrop instrument (ThermoFisher) by detecting the absorbance at 280 nm and calculated using the specific extinction coefficients. Each sample was measured in triplicate, and the average was recorded as the final concentration.

### Biolayer interferometry assay (BLI)

The kinetic assays of spike proteins with monomeric hACE2 receptor or antibodies were performed on an Octet R8 instrument (Sartorius) using standard parameters.

Briefly, the protein A biosensors (Sartorius, Cat# 29127557) were pre-incubated in assay buffer (PBS pH 7.4, 0.05% Tween20) for 15 min. Then, the biosensors were equilibrated and loaded with antibodies at a concentration of 5 mg/L. After a second baseline, the biosensors were incubated with a concentration gradient of spike proteins for 100 seconds, followed by a 200-second dissociation phase. At the end of a full association-dissociation round, the biosensors were regenerated with 10 mM glycine buffer pH 1.5. The signal data was processed using Octet Analysis Studio 12.2.0.20 (Sartorius). Curves were aligned at the baseline and blanked with the control signal. The processed curves were globally fitted using a 1:1 binding model to calculate kinetic parameters.

For the hACE2 assay, the protein was firstly biotinylated using the Sulfo-NHS-LC-LC-biotin biotinylation kit (ThermoFisher, Cat# 21338). Then SA biosensors (Sartorius, Cat# 54070491) were used to capture the biotinylated hACE2, and the following steps were similar to the antibody assays.

### Pseudovirus production

SARS-CoV-2 wild type and variant pseudoviruses were generated as described previously, with minor modifications. Briefly, HEK293T cells were grown to 70-80% confluency before co-transfection with the pCMV14 expression vector encoding either SARS-CoV-2 wild type or variant S gene, and a luciferase reporter plasmid (pNL4-3-R-E-luciferase, gifted by Dr. Wanbo Tai) at a ratio of 1:1 in Opti-MEM medium using polyethylenimine. After 5 h, the cell supernatant was replaced with fresh DMEM medium, and the cells were cultured for an additional 48 h at 37 °C in an atmosphere comprising 5% CO_2_. Pseudoviruses secreted into the supernatant were collected by centrifugation at 1000 × *g* for 10 min, filtered through a 0.45 µm pore-size membrane, and stored at −80 °C. To determine the SARS-CoV-2 pseudovirus titer, viral stocks were serially diluted2-fold with DMEM and added to 1.5 × 10^4^ hACE2-293T cells per well in 96 well tissue culture plates. After incubation for 48 h at 37 °C in an atmosphere comprising 5% CO_2_, cell supernatants were removed, 1× lysis buffer containing luciferase substrate (75 µL/well) was added to the plates, and shaken at 40 rpm for 10 min at room temperature. Cell lysates were transferred into luminometer plates (Corning, Cat# 3917). Relative luciferase activity was measured using a Synergy Neo2 Hybrid Multi-Mode Reader (BioTek, USA)

### Pseudovirus-based neutralization assay

Pseudovirus-based neutralization assays were performed by incubating serial dilutions of mAbs with SARS-CoV-2 wild type and variant pseudoviruses and calculated based on the reduction of luciferase activity. Briefly, 1.5 × 10^4^ hACE2-293T cells per well were seeded into a 96-well plate. Purified mAbs were serially 4-fold diluted in duplicate wells with completed DMEM medium to produce a concentration gradient ranging from 10 mg/mL to 0.61 µg/L, mixed with an equal volume of titrated SARS-CoV-2 wild type or mutated pseudovirus, and incubated at 37 °C for 2 h. The mAb and pseudovirus mixture was added to the cultured cells and incubated for an additional 48 h, after which the luciferase activity was measured as described.^64^

The IC50 was expressed as the dilution at which the relative luciferase units were reduced by 50% compared with the cells infected with pseudovirus without antibodies after subtraction of the background in the control groups with mock-infected cells. The IC50 values were calculated using nonlinear regression.

### In vitro assembly of SARS-CoV-2 spike protein complex with ACE2 or antibodies

Spike protein was first added into an Eppendorf tube with 500 µL of PBS to assemble the receptor-ligand or antibody-antigen complex. Then, ACE2 or antibodies were added to the tube at a 3-fold molar excess to the spike protein to guarantee complete binding. The assembly mixture was incubated at room temperature for 15 min and centrifuged at 18,000 × *g* and 4 °C for 5 min before application to the SEC column.

### Size-exclusion chromatography (SEC)

The samples were centrifuged at 18,000 × *g* for 5 min at 4 °C to remove debris before application to the Superdex200 increase 10/300GL SEC (Cytiva, Cat# 28990944) column on an ÄKTA pure25M instrument (GE healthcare). Pre-filtered PBS was used as the running buffer, and after each round of sample processing, the SEC column was equilibrated with 1 column volume of the running buffer to maintain a steady baseline.

### Statistical analysis

All statistical analyses are described in the corresponding methods sections and indicated in the figure legends. Octet Discovery Studio12.0 (Sartorius) was used to process data from the kinetic assay. GraphPad Prism 9.0 software was used to process the pseudovirus neutralization assay data.

## Supplementary information

Supplemental information

## Data Availability

All data is available in the manuscript or the supplementary materials. The code of Rosetta FastRelax and flexddG used in this study was uploaded to Github: https://github.com/bigtaotao/covid_scripts.git. All the structural representations were rendered using ChimeraX-1.1.1 or PyMOL. The protein complex structures used in this study are available at PDB (Accession number: 7KZB, 7K45, 7JX3, 7CM4, 6XDG, 7BWJ, 7L5B, 7C01, 7JMW, 6M0J, 7L0N). Under reasonable request, the experimental data could be accessed through research data deposit (RDD) with RDD number RDDB2021176511.
