# Supplementary material for: Parallel profiling of antigenicity alteration and immune escape of SARS-CoV-2 Omicron and other variants
Source: Signal Transduct Target Ther. 2022 Feb 8;7:42. doi: 10.1038/s41392-022-00910-6 (PMC8826850; doi:10.1038/s41392-022-00910-6)
Supplement: Supplementary file 1 — Supplemental information [file 41392_2022_910_MOESM1_ESM.docx]

Supplementary Materials for

**Parallel profiling of antigenicity alteration and immune escape of SARS-CoV-2 Omicron and other variants**

Cong Sun ^1^*, Yin-Feng Kang ^1^*, Yuan-Tao Liu ^1^, Xiang-Wei Kong ^1^, Hui-Qin Xu ^2^, Dan Xiong ^3^, Chu Xie ^1^, Yi-Hao Liu ^4,5,6^, Sui Peng ^4,5^, Guo-Kai Feng ^1†^, Zheng Liu ^2†^, Mu-Sheng Zeng ^1†^

Correspondence to [zengmsh@sysucc.org.cn](mailto:zengmsh@sysucc.org.cn)

**This PDF file includes:**

Materials and Methods

Figures. S1 to 6

**Materials and Methods**

Computational interface analysis

We used flexddG to detect the impact of mutations on the affinity of the interface between the RBD and receptor ACE2 or neutralizing antibodies. FlexddG is a program developed to evaluate protein-protein interfaces based on the Rosetta modeling suite (*58*). It uses "backrub" to generate a collection of wild-type or mutant models. By torsion minimization, side-chain repacking and averaging of model energy, the interface ΔΔG was calculated to determine the alteration of interfacial free energy and further assist in evaluating the affinity of the designated protein complex after the specified mutation. Firstly, we used Rosetta FastRelax to preprocess the input structures, and for each protein complex, the structure with the lowest energy out of the 15 relaxed models was selected for the next step. Then, FlexddG was used to calculate the ΔΔG of the relaxed structure with the specified mutation. The average ΔΔG value of 35 models generated by flexddG was used as the final result for each structure with a given mutation. To display the protein complex interface, the relaxed structure with the lowest energy was selected as the WT structure, and the mutated structure with the lowest energy generated by flexddG was selected as the Mut structure.

Plasmid construction

The sequences encoding spike (S) protein with a 19 amino acid deletion at the C-terminus from SARS-CoV-2 wild type (GenBank: MN985325.1) Alpha variant/MILK-9E05B3/2020 (Lineage: B.1.1.7; GISAID accession ID: EPI_ISL_601443), Beta variant/NHLS-UCT-GS-1067/2020 (Lineage: B.1.351, GISAID accession ID: EPI_ISL_700428), Gamma variant/IC-0561/2021 (Lineage: P.1; GISAID accession ID: EPI_ISL_792680), Delta variant/MP-NCDL-2509230/2020 (Lineage: B.1.617.2; GISAID accession ID: EPI_ISL_2461258), Kappa variant/ CNRST-IND2-2021/2021 (Lineage: B.1.617.1, GISAID accession ID: EPI_ISL_1719097), Lambda variant/ UPCH_cov0463/2021 (Lineage: C.37, GISAID accession ID: EPI_ISL_2158693) and Omicron variant/Rega-20174/2021 (Lineage: B.1.1.529, GISAID accession ID: EPI_ISL_6640916) were codon-optimized and synthesized (GenScript). The single-mutation variants of the spike protein based on the wild type were generated by PCR using the QuickChange site-directed mutagenesis kit (Vazyme, C113-01) following the manufacturer's instructions. The plasmids encoding wild-type or variant spike protein fused with an N-terminal Kozak sequence and C-terminal 3X flag tag were cloned into the mammalian expression vector pCMV14 using the CE-II cloning system. The wild type and variants of SARS-CoV-2 HexaPro constructs (residue 16-1138) were produced as previously reported ^63^. Briefly, the four synthetic spike genes were used for pseudovirus production as a PCR template to generate the HexaPro constructs with proline residues substituting F817, A892, A899, A942, K986, and V987, the GSAS amino acid sequence replacing the furin cleavage site (residues 682-685), the addition of a flexible linker (GSAS), and a T4 foldon trimerization motif at the C-terminus. The sequence encoding the RBD of the SARS-CoV-2 prototype (wild type spike residues 319-541) was also synthesized (GenScript). The monomeric hACE2 (residue 19-615) was produced as previously described ^64^. All SARS-CoV-2 HexaPro spike protein(*59*) used for protein production were fused with a tissue plasminogen activator (TPA) signal at the N-terminus, HRV3C protease recognition site, octa-histidine tag, and Twin-Strep-tag at the C-terminus, and then cloned into the mammalian expression vector VRC8405 (gifted by Dr. Gary J. Nabel).

The heavy and light chain sequences of 14 potent neutralizing monoclonal antibodies (mAbs), including REGN-10933, Regdanvimab, S2-E12, COVA1-16, S2-H14, S2-M11, CB6, IgG1-ab1, P2B-2F6, CR3022, COV2-2196, 4A8, Fab 2-15, and REGN-10987 tested in this study were retrieved from the National Center for Biotechnology Information (NCBI) and Protein Data Bank (PDB, codon-optimized, synthesized (GenScript), and cloned into the antibody expression vector.

Protein expression and purification

Plasmids encoding SARS-CoV-2 HexaPro and hACE2 were mixed with polyethylenimine (Polysciences, Cat# 24765) at a weight ratio (w:w) of 1:3 in serum-free Union 293 medium and used to transiently co-transfect suspension Expi293F cells. After six days, the cell culture supernatant was harvested by centrifugation, filtered through a 0.22 µm pore-size vacuum membrane, and applied to Ni Sepharose excel resin (Cytiva, Cat# 17371201). For the purification of SARS-CoV-2 HexaPro wild type and variants, the resin was washed with a buffer composed of 50 mM HEPES pH8.0, 300 mM NaCI, 30 mM imidazole, 5% glycerol, and 0.02% NaN_3_, and eluted with a buffer composed of 50 mM HEPES pH8.0, 300 mM NaCI, 500 mM imidazole, 5% glycerol, and 0.02% NaN_3_. For the purification of SARS-CoV-2 RBD wild type, its variants, and hACE2, the resin was washed with a buffer composed of 50 mM HEPES pH7.4, 300 mM NaCI, 30 mM imidazole, 0.02% NaN3, and eluted with a buffer composed of 50 mM HEPES pH7.4, 300 mM NaCI, 500 mM imidazole, and 0.02% NaN_3_. The eluted proteins were concentrated using 10 kDa MWCO Amicon Ultra centrifugal filters (Merck Millipore, Cat# UFC901096). The protein of interest was further purified by size-exclusion chromatography using a Superose 6 Increase 10/300 GL column (Cytica, Cat# 17517201) in phosphate-buffered saline (PBS, pH7.4), aliquoted, and stored at -80 °C until further use.

To produce the nAbs as described above, plasmids encoding the heavy and light chain were used at a weight ratio (w:w) of 5:6 to co-transfect suspension Expi293F cells using polyethylenimine as above. After five days, the cell supernatant containing mAbs was harvested, loaded onto protein A resin (GenScript), and eluted with glycine buffer at pH 3.0. The antibodies were further purified by size-exclusion chromatography using a Superose 6 Increase 10/300 GL column in PBS, pH7.4. Finally, the antibodies were aliquoted and stored at -80 °C. Protein concentrations were measured using the BCA method.

Cell lines

HEK293T cells were obtained from ATCC (CRL-3216) and cultured in Dulbecco's minimal essential medium (DMEM) supplemented with 10% heat-inactivated fetal bovine serum (FBS) and 1%(v/v) penicillin-streptomycin. Human angiotensin-converting enzyme 2 (ACE2) stable-expressing HEK293T cells (hACE2-HEK293T) were derived from HEK293T cells by transduction with a lentiviral vector encoding the human ACE2 gene. Suspension Expi293F cells were obtained (ThermoFisher, Cat# A14527) and grown in serum-free Union 293 medium (Union, Cat# UP1000) with shaking at 120 rpm and 37°C in a humidified atmosphere comprising 5% CO_2_. All cell lines in this study were confirmed to be free of mycoplasma contamination using MycAway™ Treatment (1000×) Mycoplasma Elimination Reagent (Yeasen, Cat# 40607ES03).

Protein quantification and storage

The protein concentrations were determined using a NanoDrop instrument (ThermoFisher) by detecting the absorbance at 280nm and calculated using the specific extinction coefficients. Each sample was measured in triplicate, and the average was recorded as the final concentration.

Biolayer interferometry assay (BLI)

The kinetic assays of spike proteins with monomeric hACE2 receptor or antibodies were performed on an Octet R8 instrument (Sartorius) using standard parameters.

Briefly, the protein A biosensors (Sartorius, Cat# 29127557) were pre-incubated in assay buffer (PBS pH 7.4, 0.05% Tween20) for 15 min. Then, the biosensors were equilibrated and loaded with antibodies at a concentration of 5 mg/L. After a second baseline, the biosensors were incubated with a concentration gradient of spike proteins for 100 seconds, followed by a 200-second dissociation phase. At the end of a full association-dissociation round, the biosensors were regenerated with 10 mM glycine buffer pH 1.5. The signal data was processed using Octet Analysis Studio 12.2.0.20 (Sartorius). Curves were aligned at the baseline and blanked with the control signal. The processed curves were globally fitted using a 1:1 binding model to calculate kinetic parameters.

For the hACE2 assay, the protein was firstly biotinylated using the Sulfo-NHS-LC-LC-biotin biotinylation kit (ThermoFisher, Cat# 21338). Then SA biosensors (Sartorius, Cat# 54070491) were used to capture the biotinylated hACE2, and the following steps were similar to the antibody assays.

Pseudovirus production

SARS-CoV-2 wild type and variant pseudoviruses were generated as described previously, with minor modifications. Briefly, HEK293T cells were grown to 70-80% confluency before co-transfection with the pCMV14 expression vector encoding either SARS-CoV-2 wild type or variant S gene, and a luciferase reporter plasmid (pNL4-3-R-E-luciferase, gifted by Dr. Wanbo Tai) at a ratio of 1:1 in Opti-MEM medium using polyethylenimine. After 5 h, the cell supernatant was replaced with fresh DMEM medium, and the cells were cultured for an additional 48 h at 37 °C in an atmosphere comprising 5% CO_2_. Pseudoviruses secreted into the supernatant were collected by centrifugation at 1000 g for 10 min, filtered through a 0.45 µm pore-size membrane, and stored at -80 °C. To determine the SARS-CoV-2 pseudovirus titer, viral stocks were serially diluted2-fold with DMEM and added to 1.5x10^4^ hACE2-293T cells per well in 96 well tissue culture plates. After incubation for 48 h at 37 °C in an atmosphere comprising 5% CO_2_, cell supernatants were removed, 1X lysis buffer containing luciferase substrate (75 µL/well) was added to the plates, and shaken at 40 rpm for 10 min at room temperature. Cell lysates were transferred into luminometer plates (Corning, Cat# 3917). Relative luciferase activity was measured using a Synergy Neo2 Hybrid Multi-Mode Reader (BioTek, USA)

Pseudovirus-based neutralization assay

Pseudovirus-based neutralization assays were performed by incubating serial dilutions of mAbs with SARS-CoV-2 wild type and variant pseudoviruses and calculated based on the reduction of luciferase activity. Briefly, 1.5x10^4^ hACE2-293T cells per well were seeded into a 96-well plate. Purified mAbs were serially 4-fold diluted in duplicate wells with completed DMEM medium to produce a concentration gradient ranging from 10 mg/mL to 0.61 µg/L, mixed with an equal volume of titrated SARS-CoV-2 wild type or mutated pseudovirus, and incubated at 37 °C for 2 h. The mAb and pseudovirus mixture was added to the cultured cells and incubated for an additional 48 h, after which the luciferase activity was measured as described^64^.

The IC50 was expressed as the dilution at which the relative luciferase units were reduced by 50% compared with the cells infected with pseudovirus without antibodies after subtraction of the background in the control groups with mock-infected cells. The IC50 values were calculated using nonlinear regression.

*In vitro* assembly of SARS-CoV-2 spike protein complex with ACE2 or antibodies

Spike protein was first added into an Eppendorf tube with 500 µL of PBS to assemble the receptor-ligand or antibody-antigen complex. Then, ACE2 or antibodies were added to the tube at a 3-fold molar excess to the spike protein to guarantee complete binding. The assembly mixture was incubated at room temperature for 15 min and centrifuged at 18000g and 4°C for 5 min before application to the SEC column.

Size exclusion chromatography (SEC)

The samples were centrifuged at 18000g for 5 min at 4°C to remove debris before application to the Superdex200 increase 10/300GL SEC (Cytiva, Cat# 28990944) column on an ÄKTA pure25M instrument (GE healthcare). Pre-filtered PBS was used as the running buffer, and after each round of sample processing, the SEC column was equilibrated with 1 column volume of the running buffer to maintain a steady baseline.

Statistical analysis

All statistical analyses are described in the corresponding methods sections and indicated in the figure legends. Octet Discovery Studio12.0 (Sartorius) was used to process data from the kinetic assay. GraphPad Prism 9.0 software was used to process the pseudovirus neutralization assay data.

**Supplementary Figures and Legends**

**
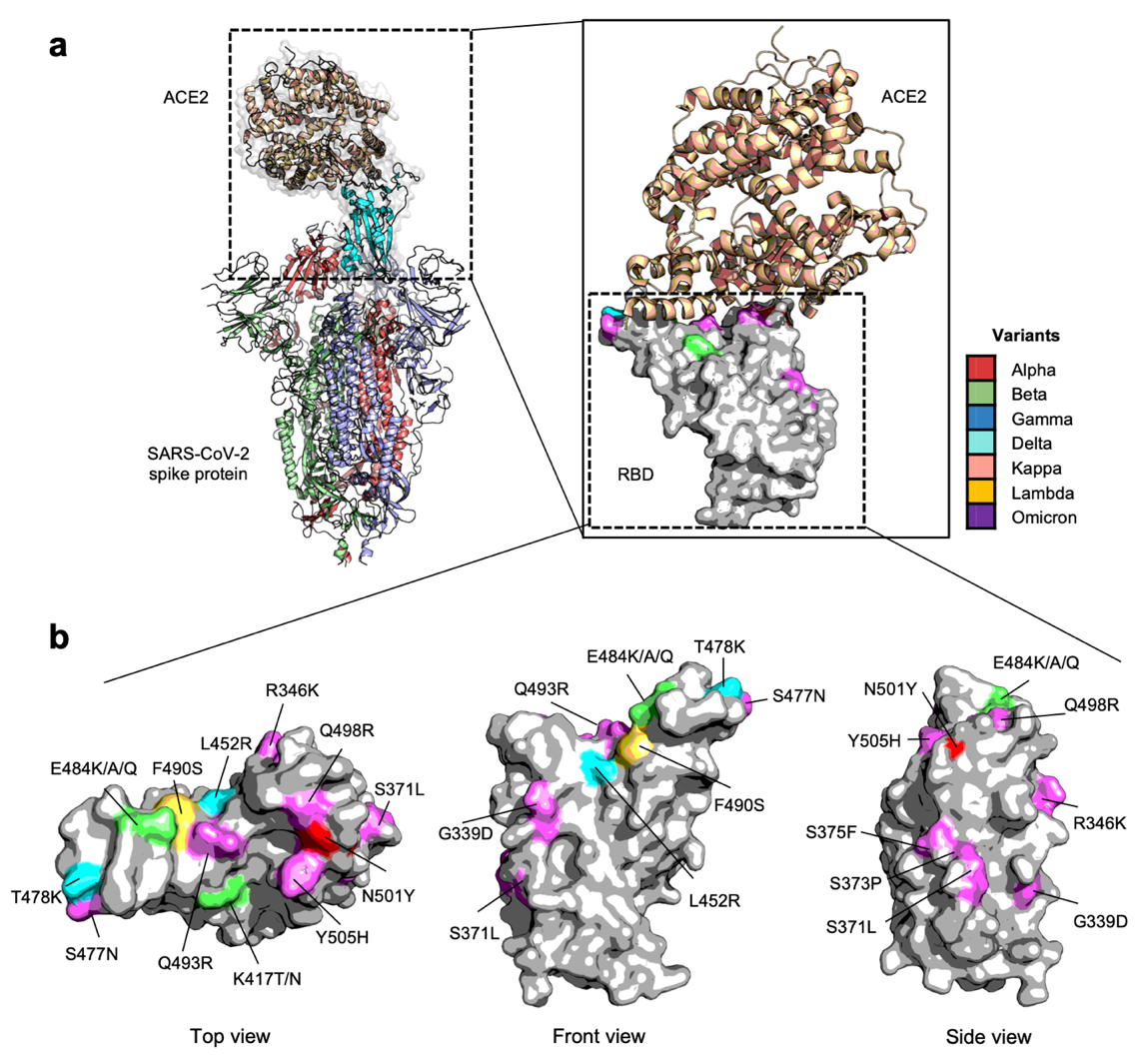
**

**Figure. S1. Structural overview of SARS-CoV-2 spike protein and the mutations from variants.**

1. Structural representation of the SARS-CoV-2 spike protein – ACE2 complex (PDB: 7DF4). The spike protein trimer and ACE2 are colored by chain.
2. The footprint of mutations located on RBD from different SARS-CoV-2 variants (PDB: 6M0J). Mutations are marked besides, and the variants color the corresponding surfaces.

**
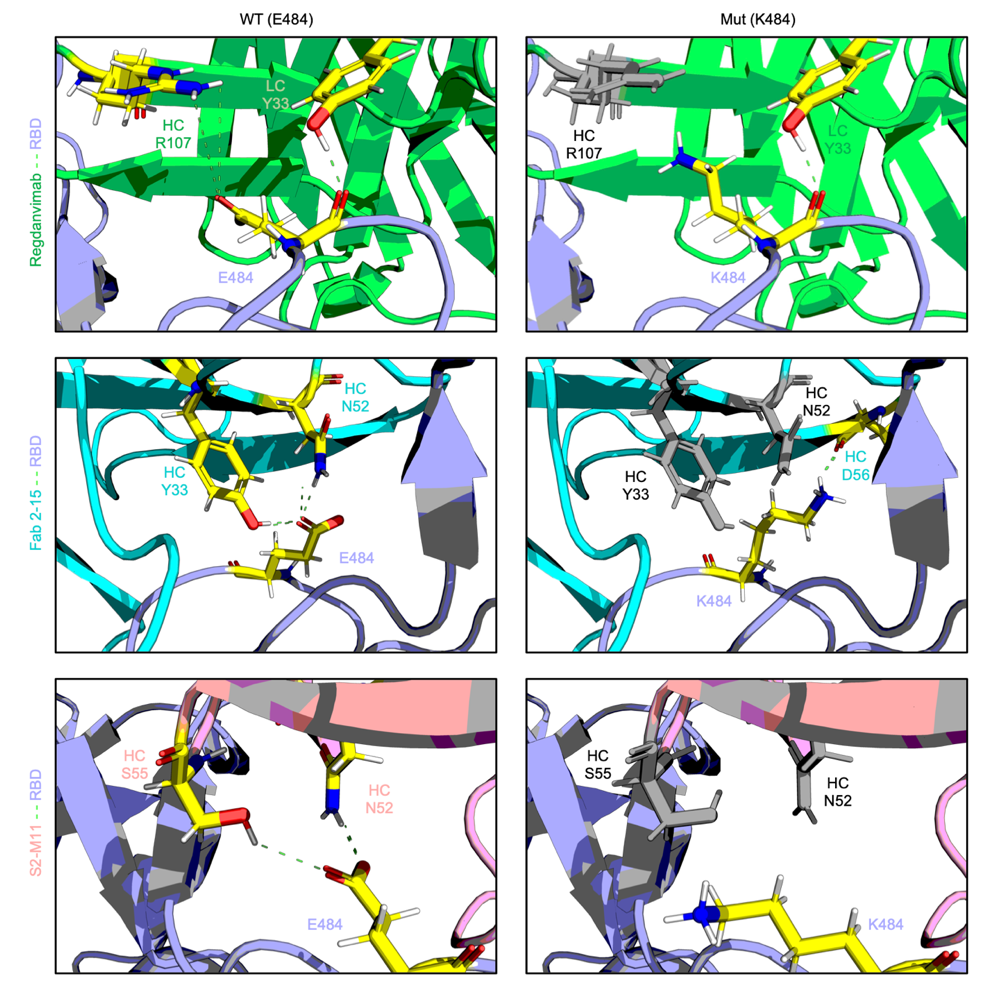
**

**Figure. S2. The E484K mutation impaired the interaction of the neutralizing antibodies Regdanvimab, Fab 2-15, and S2-M11 with the RBD.**

E484, an acidic amino acid, can establish polar connections with polar residues of binding antibodies. The conversion to K484, a basic amino acid, significantly altered the interaction between residues by reducing the number of hydrogen bonds. Critical interacting residues are displayed as sticks, colored by atom, and marked adjacently according to chain color, and hydrogen bonds are displayed as green dashed lines. Residues of the antibody chains that lost hydrogen bond connections to K484 of RBD are shown in grey.

**
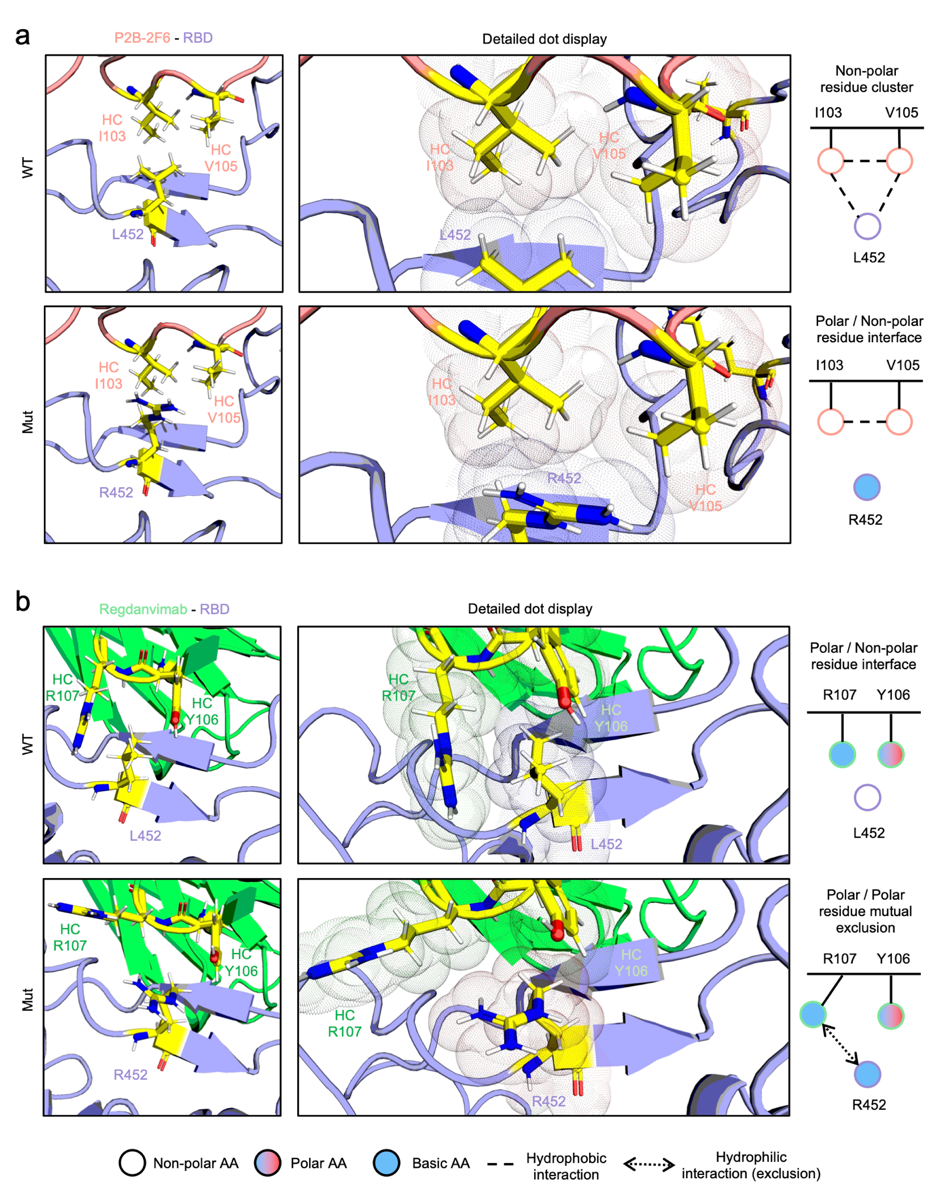
**

**Figure. S3. The L452R mutation from the delta variant manifested a distinct behavior in interrupting the RBD interaction with the neutralizing antibodies P2B-2F6** **(A) and Regdanvimab (B).**

The minor ΔΔG increase of P2B-2F6 and Regdanvimab in the presence of the L452R mutation in the RBD suggests a different mechanism through which this mutation affects the antibody-antigen interaction compared to E484K. The L452R mutation leads to a transition from a non-polar residue to a basic residue, causing a disruption in non-polar interactions with P2B-2F6 or introducing polar exclusion in Regdanvimab. Critical interacting residues are displayed as sticks, colored by atom, and marked adjacently according to chain color. Hydrogen bonds are displayed as green dashed lines. Diagrams of interaction patterns for each structure are also shown.


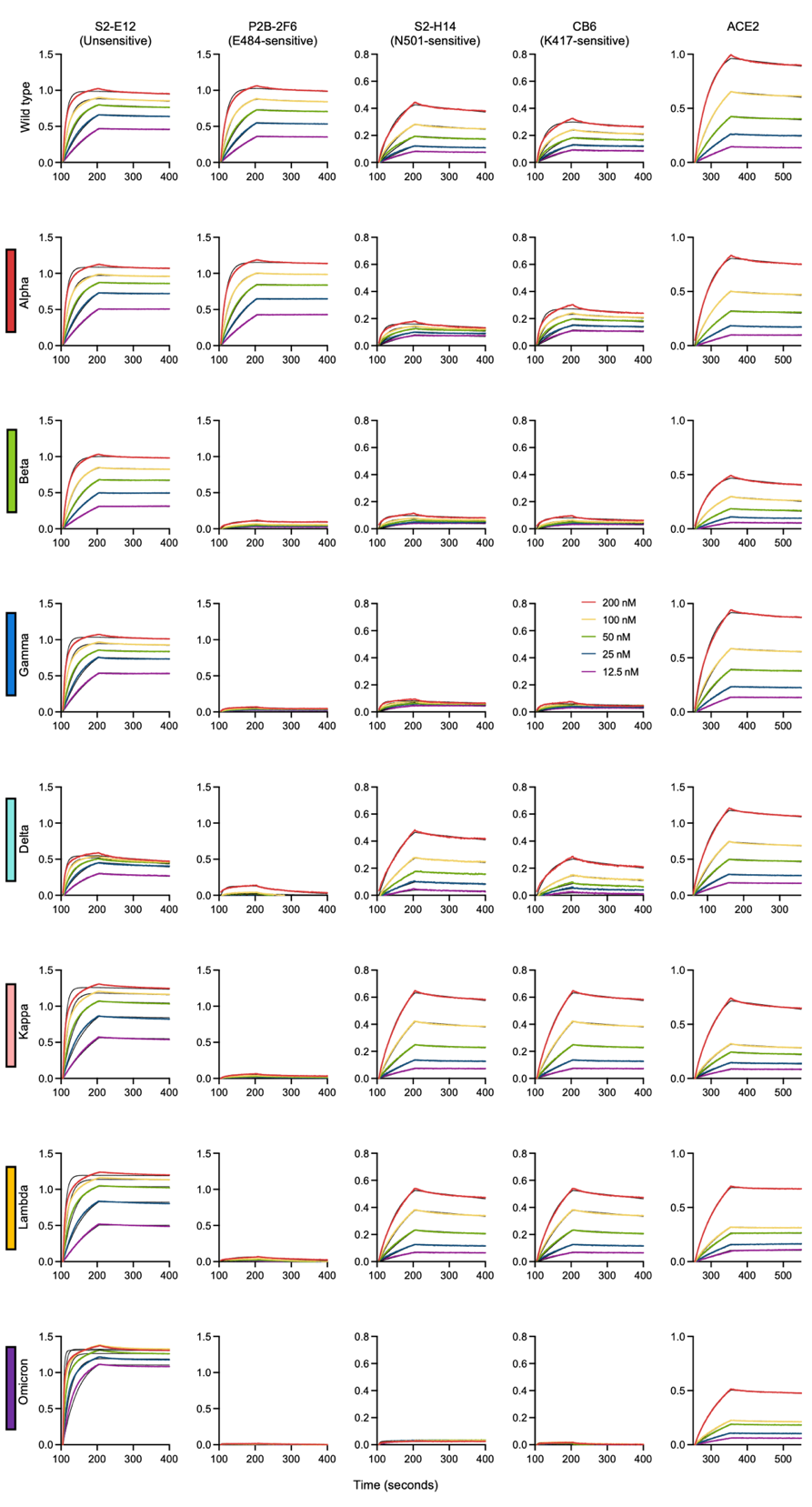


**Figure. S4. Biolayer interferometry results of neutralizing antibodies or receptor ACE2 binding to SARS-CoV-2 spike protein variants.**

The Y-axis of each chart is the binding signal (nm). Colored lines represent the binding signal of spike protein at different concentrations during the association and dissociation phases. Fitted curves are displayed as black lines.

**
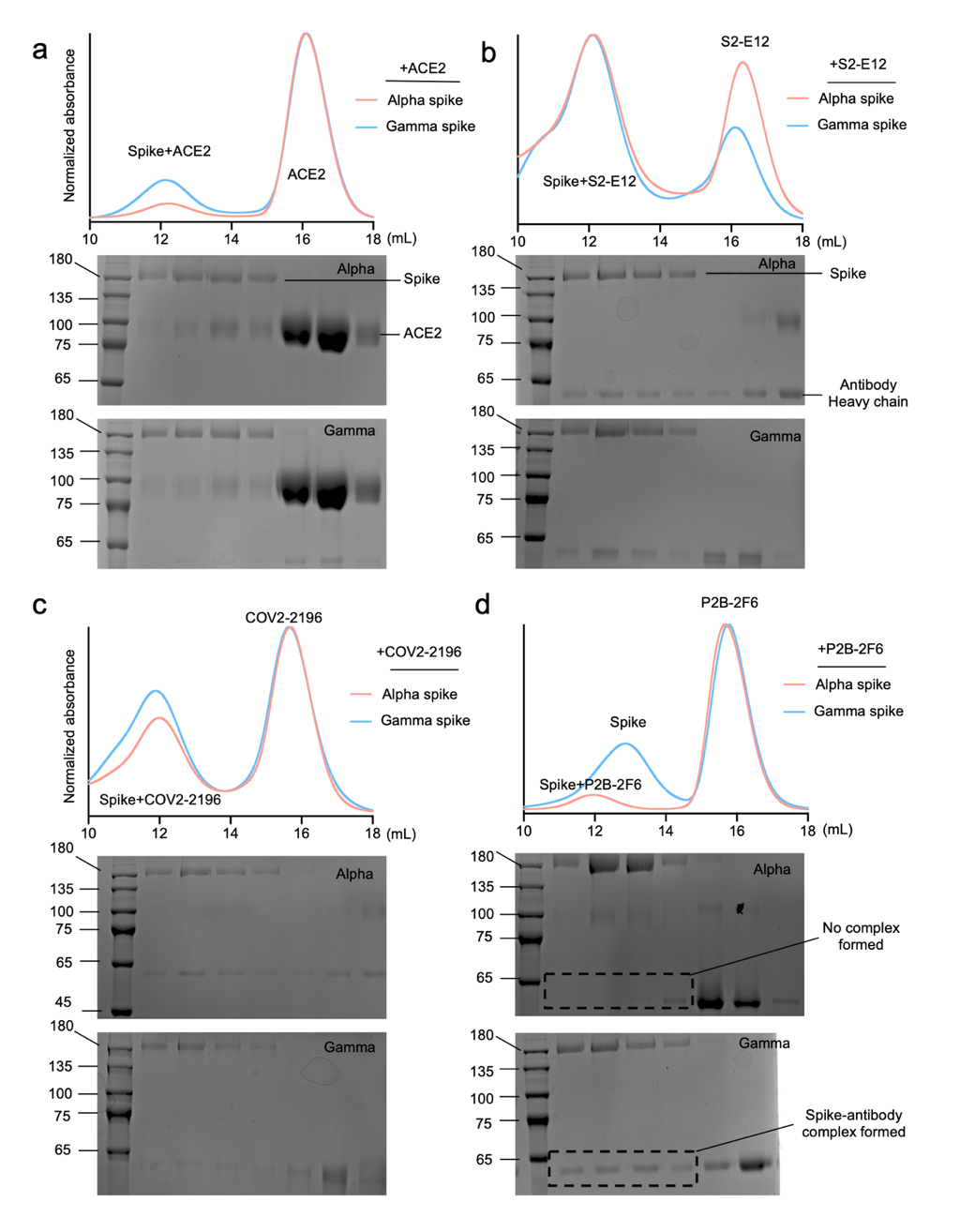
**

**Figure. S5. *In vitro* assembly of SARS-CoV-2 spike Alpha or Gamma variants in complex with the receptor ACE2 (A) or neutralizing antibodies S2-E12 (B), COV2-2196 (C), and P2B-2F6 (D).**

Following incubation of the two components, the spike-ACE2 or spike-antibody complexes could be separated by size exclusion chromatography (SEC) and further analyzed by reducing SDS-PAGE. The SEC chromatography and the SDS-PAGE gel images are aligned by the elution sequence. Different variants are colored differently and labeled adjacently according to the SEC chromatography. The bands visible on the SDS-PAGE gel are marked adjacent to the gel image.

**
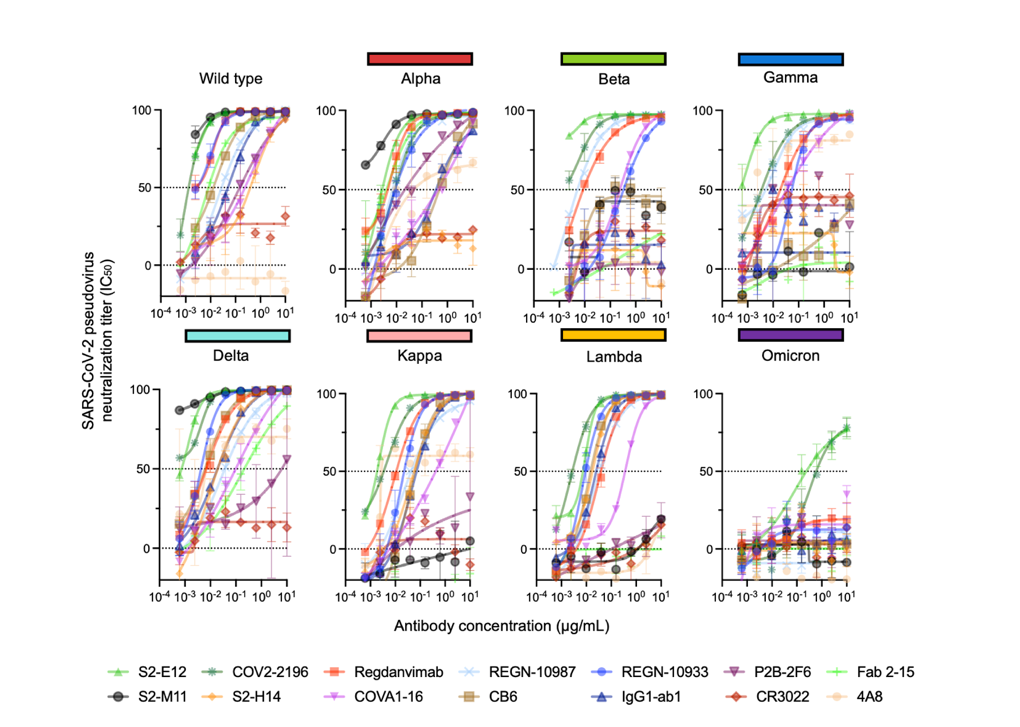
**

**Figure. S6. Neutralization of SARS-CoV-2 pseudovirus variants by the neutralizing antibody panel.**

The infection of 293T cells with the pseudoviruses induces expression, used as the indicator of cell infection. For each antibody, the neutralization rate was calculated by dividing the luciferase signal in the presence of the indicated antibody concentrations with the mean signal in the absence of the antibody. Duplicate experiments' mean neutralization rate and standard deviation (SD) indicate different antibodies with different lines. Non-linear fitting was used to calculate the IC_50_.
